# Concentrations of toxic metals and essential trace elements vary among individual neurons in the human locus ceruleus

**DOI:** 10.1371/journal.pone.0233300

**Published:** 2020-05-19

**Authors:** Roger Pamphlett, Rachel Mak, Joonsup Lee, Michael E. Buckland, Antony J. Harding, Stephen Kum Jew, David J. Paterson, Michael W. M. Jones, Peter A. Lay

**Affiliations:** 1 Discipline of Pathology, Sydney Medical School, Brain and Mind Centre, The University of Sydney, Sydney, New South Wales, Australia; 2 Department of Neuropathology, Royal Prince Alfred Hospital, Brain and Mind Centre, Sydney, New South Wales, Australia; 3 School of Chemistry and Sydney Analytical, The University of Sydney, Sydney, New South Wales, Australia; 4 Australian Synchrotron, Clayton, Victoria, Australia; Chinese Academy of Sciences, CHINA

## Abstract

**Objective:**

Damage to locus ceruleus neurons could play a part in the pathogenesis of neurodegenerative disorders such as Alzheimer’s disease, Parkinson’s disease, and multiple sclerosis because of impairment of the blood-brain barrier and enhanced neuroinflammation. The locus ceruleus has connections throughout the brain and spinal cord, so the characteristic widespread multifocal pathology in these disorders could be due to damage to different subsets of locus ceruleus neurons. Previous studies have shown that only certain locus ceruleus neurons accumulate the neurotoxic metal mercury. To find out if concentrations of other toxic metals or of essential trace elements also vary between individual locus ceruleus neurons, we used synchrotron X-ray fluorescence microscopy on frozen sections of locus ceruleus neurons taken from people with multiple sclerosis, in whom the locus ceruleus is structurally intact.

**Materials and methods:**

Paraffin embedded sections containing the locus ceruleus from seven people with multiple sclerosis were stained with autometallography that demonstrates accumulations of mercury, silver and bismuth. These were compared to maps of multiple elements obtained from frozen sections of locus ceruleus neurons from the same people using X-ray fluorescence microscopy. Neurons in the anterior pons from three of these donors were used as internal controls.

**Results:**

Autometallography staining was observed in scattered locus ceruleus neurons from three of the seven donors. X-ray fluorescence microscopy showed variations among individual locus ceruleus neurons in levels of mercury, selenium, iron, copper, lead, bromine, and rubidium. Variations between donors of locus ceruleus neuronal average levels of mercury, iron, copper, and bromine were also detected. Anterior pons neurons contained no mercury, had varied levels of iron, and had lower copper levels than locus ceruleus neurons.

**Conclusions:**

Individual human locus ceruleus neurons contain varying levels of toxic metals and essential trace elements. In contrast, most toxic metals are absent or at low levels in nearby anterior pons neurons. The locus ceruleus plays a role in numerous central nervous system functions, including maintaining the blood-brain-barrier and limiting neuroinflammation. Toxic metals, or alterations in essential trace metals within individual locus ceruleus neurons, could be one factor determining the non-random destruction of locus ceruleus neurons in normal aging and neurodegenerative diseases, and subsequently the sites of the widespread multifocal central nervous system pathology in these disorders.

## Introduction

Most central nervous system (CNS) noradrenaline comes from the locus ceruleus (LC), and LC neurons make direct contact with adrenoreceptors on neurons, astrocytes, oligodendrocytes, microglia and capillaries [1–4]. Noradrenaline has both excitatory and inhibitory actions on neurons, it optimises various functions of astrocytes, it suppresses inflammation, mostly because of its effects on microglia, and it activates oligodendrocyte adrenoreceptors which influences the production of myelin [4,5]. The LC innervates the great majority of the CNS microvasculature and its associated astrocytic endfeet [6], with LC-derived noradrenaline playing an important part in maintaining the integrity of the blood-brain barrier [7].

Major excitatory efferents of the LC innervate CNS regions that are involved in Alzheimer’s disease (hippocampus, neocortex, basal forebrain), Parkinson’s disease (substantia nigra) and amyotrophic lateral sclerosis (brain stem and spinal motor neurons) [4]. LC neurons are damaged early in Alzheimer’s disease and Parkinson’s disease, with extensive losses later in the course of these diseases [8–12]. The topographical distribution of cell loss in the LC differs, and is non-random, in normal aging [13], Alzheimer’s disease [14], and Parkinson’s disease [15]. The types of pathology differ as well, with a loss of LC neurons in Alzheimer’s [8] and Parkinson’s disease [14], neuronal shrinkage in amyotrophic lateral sclerosis [16], and subtle gliosis in multiple sclerosis [17,18]. The reasons for these topographical and pathological variations within the LC remain unknown.

**Fig 1:**
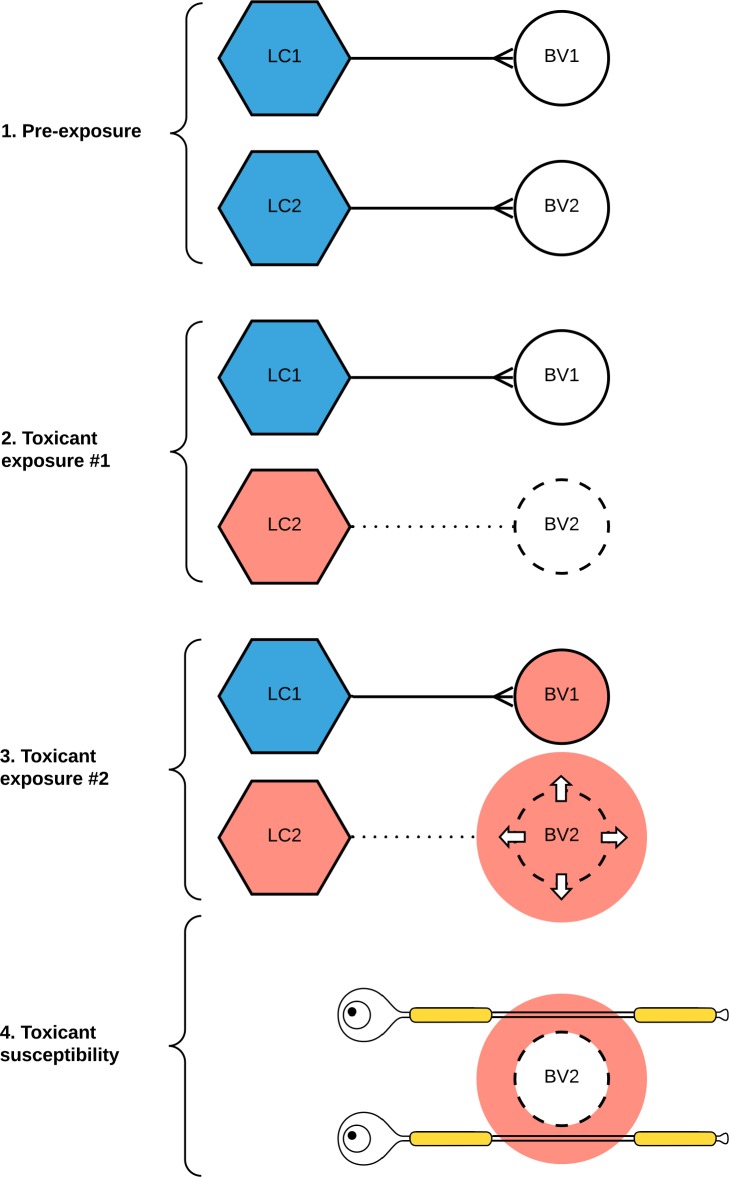
Hypothesis for selective locus ceruleus (LC) neuronal toxicity causing focal nervous system pathology, using multiple sclerosis as an example. (**1**) Normal LC neurons (blue) maintain the integrity of the blood-brain barrier of small blood vessels (BV) in the central nervous system via their output of noradrenaline. (**2**) On initial exposure to a circulating toxicant, the toxicant is taken up selectively by some LC neurons (red) only. A subsequent decrease in noradrenaline output impairs the blood-brain barrier of blood vessels innervated by these toxicant-containing LC neurons. (**3**) On further exposure to the same (or a different) circulating toxicant, the toxicant passes through the damaged blood-brain barrier. (**4**) Depending on the type of toxicant exposure and the individual’s genetic susceptibility, tissue damage results from several pathological mechanisms, including free radical generation, neuroinflammation, or apoptosis. Here, a toxicant penetrating a damaged blood-brain barrier has induced axonal demyelination, resulting in a multiple sclerosis plaque.

The LC selectively takes up toxic metals such as mercury [19,20], which has led to the suggestion that toxicant-induced damage to LC neurons, particularly in the presence of underlying genetic susceptibilities, could lead to a variety of neurodegenerative or demyelinating disorders [21,22]. However, the presence of metal toxicants alone does not explain the topographical and pathological differences in LC neurons, or why the major neurodegenerative and demyelinating disorders affect different parts of the CNS. A previous study has shown that only certain, apparently random, LC neurons contain mercury, always with adjacent mercury-free neurons [19,20]. Differential uptake of mercury raises the possibility that a neuron-to-neuron variability in toxicant uptake could be one reason only certain neurons in the LC are damaged, which in turn could lead to widespread, multifocal, regions of the CNS being affected by disease (Fig 1).

Only a proportion of people with Alzheimer’s or Parkinson’s disease have mercury in their LC neurons [21,22], but other toxic metals or variations in essential trace elements could play a part in the pathogenesis of these and other CNS disorders. The histochemical technique of autometallography is a sensitive method of detecting mercury, silver and bismuth in individual cells [23,24], but to look for evidence of cell-to-cell variation in a large number of toxic and essential elements, a technique such as synchrotron X-ray fluorescence microscopy (XFM), that simultaneously detects multiple elements, is required [25]. Another technique that has been used to look for toxic metals in the LC, laser ablation-inductively coupled plasma-mass spectrometry [20], is not sensitive enough to measure levels of elements within individual cells. To undertake this study, human autopsy-derived brain stem tissue was needed that: (**1**) had been bisected in the mid-sagittal plane, with one side fresh-frozen and stored at -80°C for XFM, and one side fixed in formalin for neuropathological diagnosis and autometallography; and (**2**) had structurally intact LCs. Tissues fulfilling these criteria were available from a multiple sclerosis research tissue bank, so we used a combination of AMG and XFM to characterise the distribution and concentrations of toxic metals and essential trace elements in these individual human LC neurons, and compared the concentrations with nearby control neurons in the anterior pons.

## Materials and methods

### Ethics

This study, "The role of the locus ceruleus in disorders of the nervous system" (X14-029), was carried out in accordance with the ethical standards of the Human Ethics Review Committee of the Sydney Local Health District (Royal Prince Alfred Hospital Zone) and with the Declaration of Helsinki as revised in 2000. Donors to the Multiple Sclerosis Research Australia Brain Bank give signed consent for their central nervous system tissue to be used after death for ethics committee-approved research.

### Tissue samples

Tissue for the study was obtained from seven women (mean age at death 65 years) who had been diagnosed by a neurologist as having multiple sclerosis and who had pre-donated their brains and spinal cords to the Multiple Sclerosis Research Australia Brain Bank (**Table 1**). The mean clinical duration of multiple sclerosis was 29 years. Mobility before death was recorded as being either ambulant (2), impaired (2), wheelchair dependent (1) or bedbound (2). The mean interval between time of death and autopsy was 14 hours. The diagnosis of multiple sclerosis was confirmed on histological examination of brain and spinal cord tissue by a neuropathologist. All donors had secondary progressive multiple sclerosis with multiple chronic demyelinated plaques, and one (P6) also had recent plaques. Causes of death were infection (3), cardiorespiratory compromise (2), cancer (1) and choking (1).

**Table 1:** Characteristics of tissue donors and grading of locus ceruleus mercury.

Donor ID no.	Age range (years)	MS duration (years)	Post mortem interval (hours)	Regions studied	LC AMG grade
P1	70–74	38	4	LC	2+
P2	60–64	28	29	LC, AP	3+
P3	65–69	36	15	LC, AP	1+
P4	50–54	7	17	LC, AP	0+
P5	80–84	42	15	LC	0+
P6	50–54	20	13	LC	0+
P7	65–69	38	8	LC	0+

AMG: autometallography, AP: anterior pons, LC: locus ceruleus, MS: multiple sclerosis. See text for AMG grading criteria.

### Sample preparation

**Fig 2:**
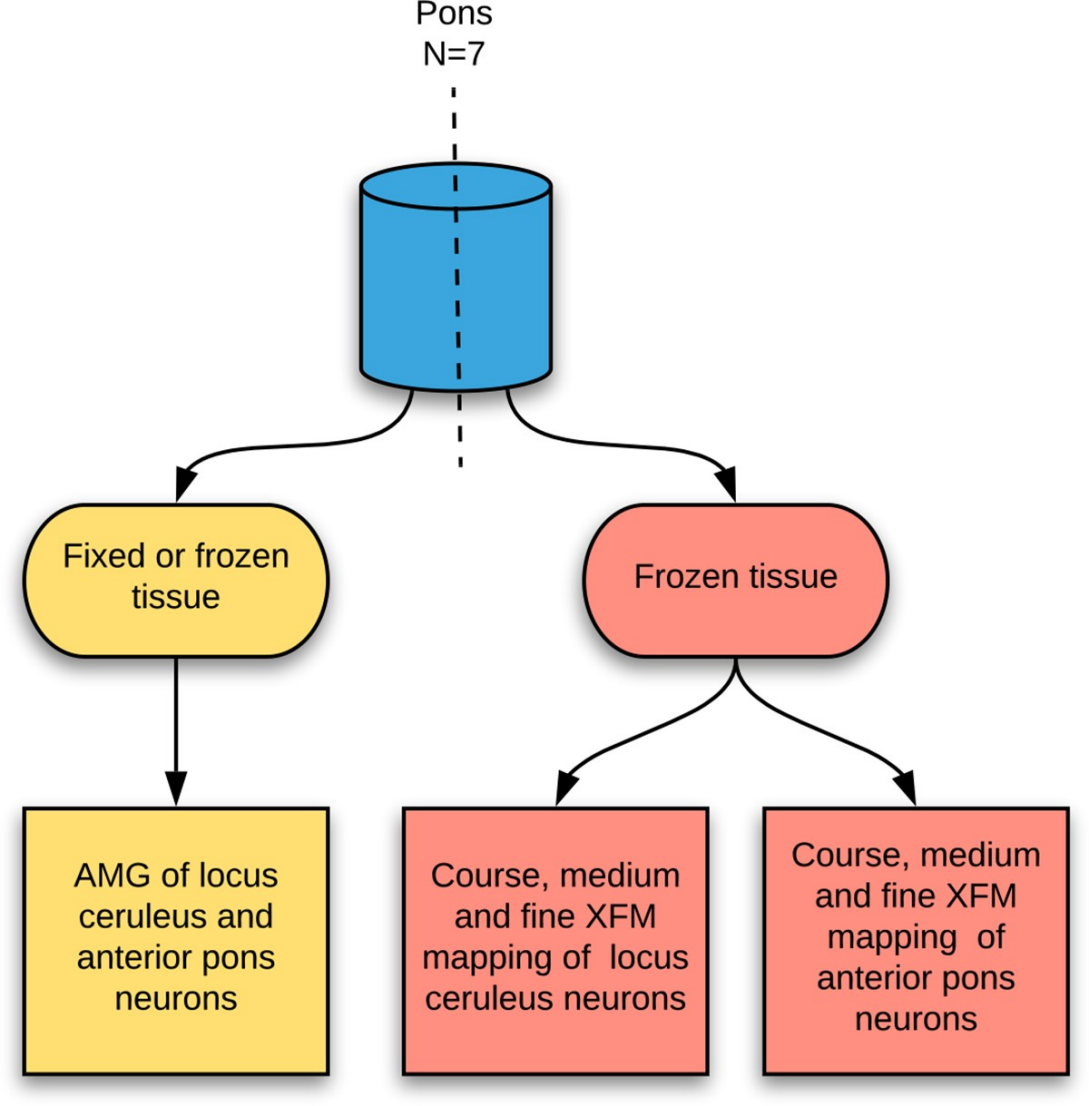
Tissue sampling. Tissues were bisected sagittally so that paired paraffin and frozen sections could be prepared from the same individual. Fixed (six pons) or frozen (one pons) sections were used for autometallography. Frozen sections from seven samples were used for XFM elemental mapping. AMG: autometallography, XFM: X-ray fluorescence microscopy.

Samples of pons containing the LC and anterior pontine neurons were bisected mid-sagittally. Half of the blocks were frozen at the time of autopsy with isopentane pre-cooled to -80°C. The other half were fixed in formalin. 10 μm-thick paraffin or frozen sections were stained with autometallography (AMG), and 10 μm-thick frozen sections examined with XFM (**Fig 2**).

### Autometallography

Briefly, sections were placed in physical developer containing gum arabic, citrate buffer, hydroquinone and silver nitrate at 26°C for 80 min in the dark, washed in sodium thiosulfate to remove unbound silver, counterstained with mercury-free hematoxylin and viewed under bright-field microscopy [26]. An AMG-positive neuron was defined as having ten or more black grains in the cytoplasm. A positive control section in each staining run was from a formalin-fixed paraffin-embedded mouse spinal cord where motoneurons contained mercury after exposure to mercuric chloride [27]. Sections stained under the protocol used here demonstrate accumulations of the sulfides or selenides of mercury, silver, and bismuth [28], with the silver-coated deposits of these three metals visible microscopically as black-staining grains. Previous elemental analysis studies using a combination of AMG and laser ablation-inductively coupled plasma-mass spectrometry indicate that in human tissues AMG is demonstrating mercury in the great majority of cases [20,29], so in this study the terms AMG staining and mercury content are used interchangeably.

The proportion of LC neurons containing AMG was graded on the percentage of positive neurons within the transverse boundary of the body of the LC (defined by the outermost neuromelanin-pigmented neurons) as 0+: no AMG neurons, 1+: 1–10% AMG neurons, 2+: 11–50% AMG neurons, and 3+: more than 50% AMG neurons. The proportion of our multiple sclerosis donors who had any AMG-stained LC neurons was compared with those of 19 females in the same age range from a previously-reported mixed clinicopathological autopsy population [20], using chi-square contingency testing with Prism v8.4 software.

### X-ray fluorescence microscopy (XFM)

All XFM experiments were conducted with cryofixed brain sections to avoid the elemental contamination and redistribution, particularly of essential trace elements, that occur on formalin fixation [30]. Frozen sections were mounted on 4×4 mm^2^ silicon nitride windows, with the regions of interest positioned within the window, and the sections freeze-dried. Imaging was performed at the XFM beamline of the Australian Synchrotron [25] using a high-performance Maia detector [31], which features a 384-element silicon diode detector array with a central aperture through which the X-ray beam passes and interacts with the sample. The fluorescence X-rays produced by the sample were captured by the detector that was 180° to the incident beam in a backscatter geometry. An advantage of this detector is that it can be brought to within fewer than 10 mm from the sample, so that a large solid angle (1.3 steradian) is subtended by the sample at the detector to maximise signal collection. Fluorescent X-ray photons were excited with a 15.8 keV X-ray beam focused with a Kirkpatrick-Baez mirror pair to a ~2 μm FWHM spot, exciting K-shell fluorescence of elements with atomic numbers from sulfur up to rubidium, along with the L-shell fluorescence of heavier elements such as mercury. X-ray fluorescence energies falling below those of the sulfur K-shell, such as phosphorus, are below the sensitivity of the detector.

Each sample was mounted on a motorised stage that moved in a two-dimensional plane orthogonal to the beam axis and enabled regions of interest within the sample to be scanned. The scanning sampling intervals combined with the beam spot size resulted in a resolution approaching 1 μm. The dwell time for the X-ray beam to interact with the sample at each pixel increased with the signal-to-noise desired. The Maia detector captured the fluorescence X-rays from a large solid angle subtended by the sample, allowing enough signal to be collected during a short dwell time, in the order of a few milliseconds or less per pixel, without observable radiation-induced damage [32]. In each region, six neurons with the largest surface areas on potassium maps were chosen for measurement of their intracellular element levels.

**Fig 3:**
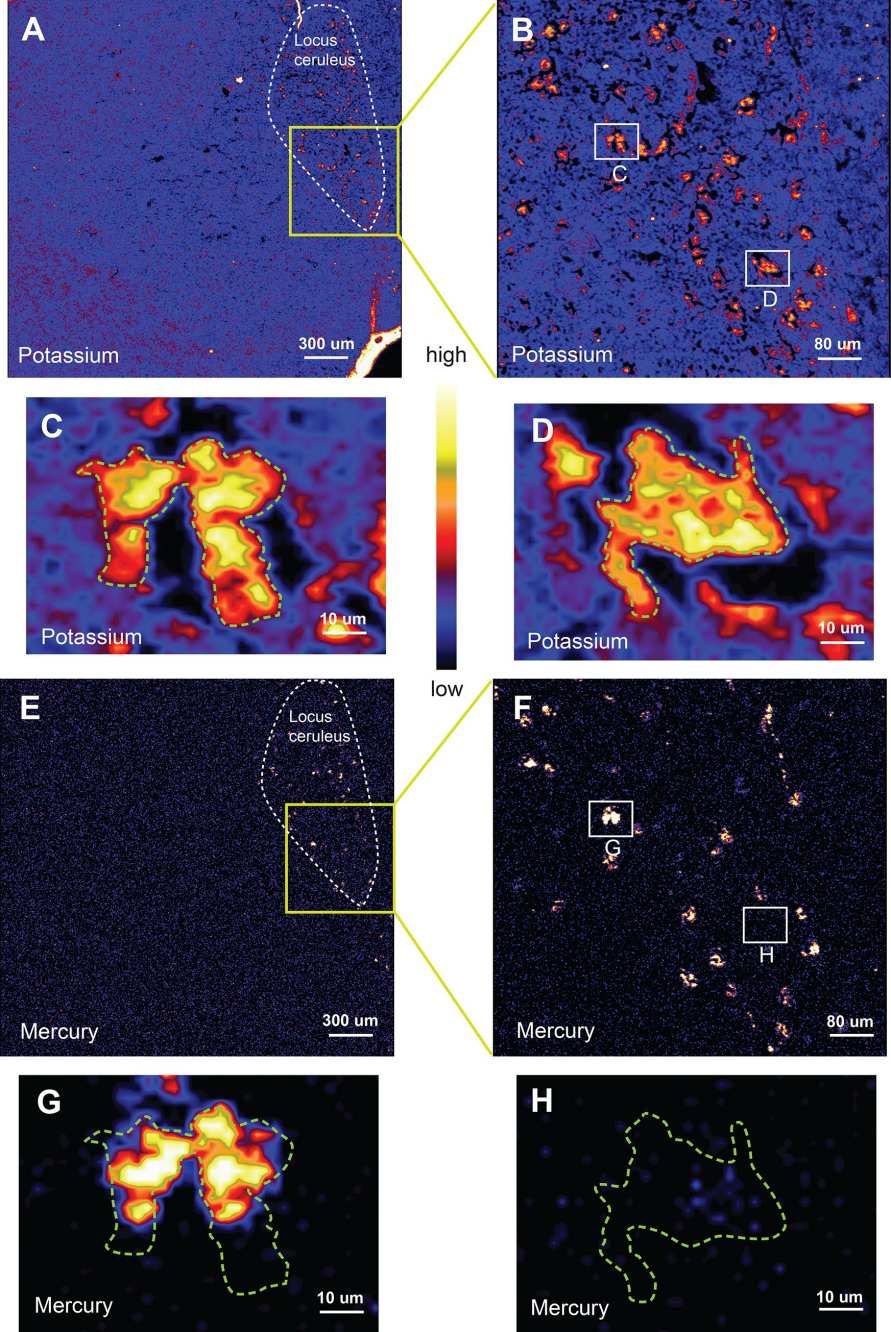
Coarse, medium, and fine-resolution XFM potassium and mercury maps of locus ceruleus (LC) neurons. All images are from the same LC neurons of donor P2. (**A**) A coarse-resolution potassium map shows multiple potassium-rich (mostly red/yellow) LC neurons at the top right of the section (yellow streaks are artefact due to section folding or edge effect). (**B**) A medium-resolution potassium map (from the box in A) shows the distribution of neurons in the LC. Medium-resolution potassium maps of two closely-adjacent neurons (**C**) and of one other neuron (**D**) in the LC shows their cell bodies filled with potassium. (**E**) A mercury map of the same neurons shows specificity of staining in the LC at coarse-resolution and (**F**) medium-resolution. On fine-resolution mapping, parts of the adjacent neurons contain mercury (**G**) whereas the other neuron (**H**) is mercury-free. Colour bar = relative concentrations of elements in parts per million (the absolute concentrations can be viewed in the Supplementary Data Sheets).

Dynamic analysis with real-time deconvolution of X-ray fluorescence spectra was performed using GeoPIXE software, which produced a map for each element showing its concentration at each pixel (**Fig 3**), as well as an integrated fluorescence spectrum for each scan [33]. Spectra were also extracted from specific regions of interest within the scan. Peak positions in the spectrum occur at specific energies for all elements present in the region. Background contributions were eliminated by subtracting the elemental concentrations measured in areas of the silicon nitride window where there was no tissue deposition, or in empty regions created by tissue shrinkage or in the empty lumen of a blood vessel. The 10 μm section had an estimated average dry-weight density of 0.23 g/cm^3^ [34]. Concentrations were recorded as parts-per-million (ppm) assuming a uniform 10 μm thickness, and as the thickness independent measure areal density (ng/cm^2^).

To make measurements of elemental concentrations comparable between different cell cross-sections, they were normalised to the concentration of potassium, since the intracellular potassium concentration is generally uniformly distributed within the cell cytoplasm. Numerical results of concentrations of elements that varied most among LC neurons (i.e., mercury, selenium, iron, copper, lead, bromine, and rubidium), are presented in histograms. An example of the link between the mapping of the cellular elements and the construction of the histograms is given in **S1 Fig**. Complete numerical details of concentrations of these and other elements that varied little among neurons (i.e., calcium, chloride, phosphorus, sulfur, and zinc), or were not or seldom detected (i.e., gold, cobalt, chromium, manganese, nickel, platinum, and tin) are presented in **S1 Table**, together with statistics regarding concentrations averaged from multiple neurons, uncertainty estimates, and minimum detectable levels.

## Results

### Autometallography

**Fig 4:**
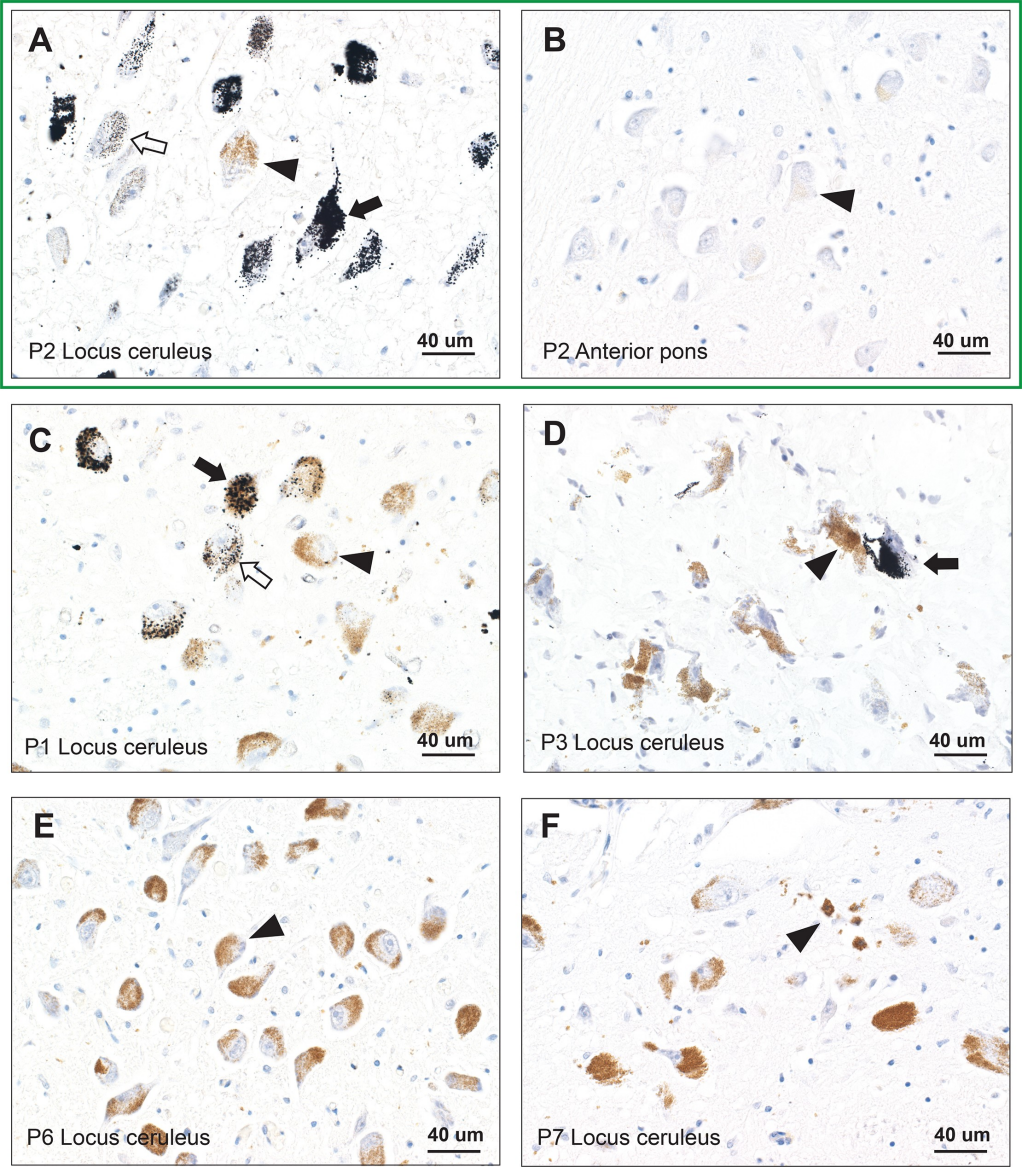
Autometallography of locus ceruleus (LC) and anterior pons neurons. Sections within the box are from the same donor. (**A**) AMG staining (black grains) is present in more than 50% of LC neurons in donor P2, and ranges from heavy staining (closed arrow), light staining (open arrow), or no staining (arrowhead) in the normally brown-stained neuromelanin-containing nearby neurons. (**B**) The paired anterior pons sample from donor P2 shows no AMG staining in neurons (e.g., arrowhead). (**C**) In donor P1, AMG staining is present in 10–50% of LC neurons, with variation of heavy (closed arrow), light (open arrow), or no (arrowhead) staining of nearby neurons. (**D**) In donor P3 (frozen section) fewer than 10% of LC neurons stain with AMG, with stained (closed arrow) and unstained (arrowhead) neurons in proximity. (**E**) Only AMG-unstained LC neurons (e.g., arrowhead) are present in donor P6. AMG and hematoxylin. (**F**) No mercury is seen on AMG staining in these LC neurons. A small collection of free and macrophage-bound neuromelanin pigment is present (arrowhead).

AMG staining was seen within LC neurons in three of the seven multiple sclerosis donors (**Table 1**). In each of these donors the proportion of AMG LC neurons varied, with some LC regions showing few, and others numerous, stained neurons, with stained and un-stained neurons often close to each other (**Fig 4**). The intracellular density of AMG in these three donors also varied among neurons, with admixtures of heavy and light staining. For example, in donor P1 more than 50% of LC neurons stained with AMG, with some neurons staining heavily, others lightly, and others not at all.

The proportion of multiple sclerosis donors who had any mercury in their LC neurons (3 out of 7, 43%) was not significantly different to the proportion (11 out of 19, 58%) of females within the same age range in a general autopsy population who had mercury in their LC (chi-square *p* = 0.68). In these general population AMG-positive females, similar variations to the multiple sclerosis individuals were observed in mercury content between nearby LC neurons [20].

LC neurons appeared normal in number and appearance in all multiple sclerosis donors, both those with and those without AMG staining. A few small foci of free and macrophage-bound pigment indicated destruction of pigmented neurons (**Fig 4**) in some individuals, a common occurrence in normal aging, especially in the rostral LC [13]. No mercury was seen in any anterior pons neurons (**Fig 4**), despite two of these multiple sclerosis donors having nearby AMG-stained LC neurons.

### Synchrotron X-ray fluorescence microscopy

**Fig 5:**
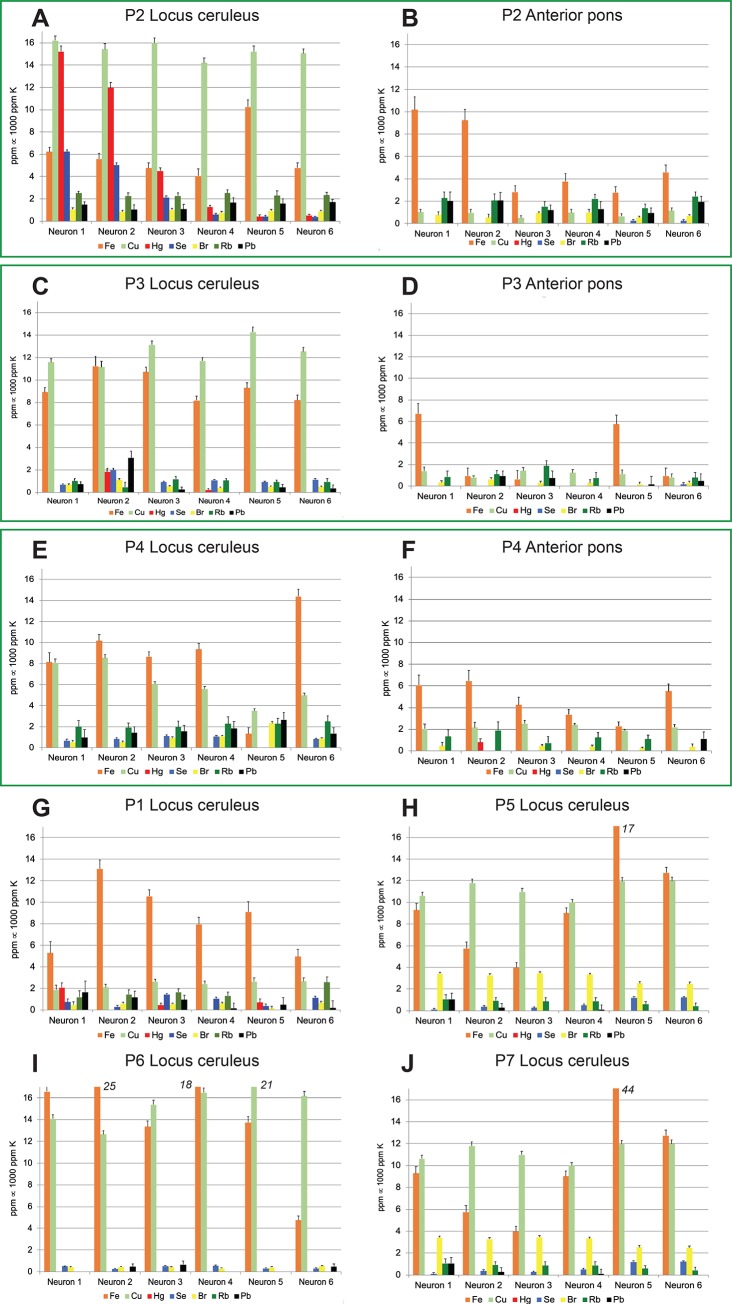
Differences in elemental composition among locus ceruleus (LC) and anterior pons neurons. Histograms within the boxes are paired from the same donor. More copper is present in LC neurons (**A**) than in anterior pons neurons (**B**); anterior pons neurons contain no mercury. More copper and iron are present in LC neurons (**C**) than in anterior pons neurons (**D**); no mercury is present in anterior pons neurons. More iron, copper and lead are present in LC neurons (**E**) than in anterior pons neurons (**F**). XFM mappings of four LC samples from P1, P5, P6 and P7 (**G, H, I, J**) show elemental differences, both among individual neurons from the same donor, and between donors. Note the high levels of iron and copper in some LC neurons.

Examination of the histograms generated by the elemental mapping show marked differences in the elemental composition among LC and anterior pons neurons (**Fig 5**). In addition, the average concentrations of several elements in LC neurons varied between different people (**Table 1**). The greatest variations were seen in the following toxic metals and essential trace elements: (**1**) *Mercury* was observed in high concentrations in LC neurons that stained positively for AMG. Mercury levels varied widely between individual LC neurons of the same person. Mercury was not detected in significant amounts in anterior pons neurons. (**2**) *Selenium* was present in most LC neurons, and at high levels (in a stoichiometric ratio of selenium to mercury of 1:1) in neurons containing high levels of mercury. (**3**) *Iron* concentrations often varied between LC neurons, with some individual LC neurons reaching high levels. Iron concentrations were higher on average in LC than in anterior pons neurons. (**4**) *Copper* concentrations varied widely in LC neurons, from undetectable to above 10 ppm, and were higher on average in the LC than in anterior pons neurons. (**5**) *Lead* was present at low levels in neurons in the LC and anterior pons, with values above 2 ppm in individual neurons only in the LC of donors P3 and P4. (**6**) *Bromine* levels less than 2 ppm were seen in many LC and anterior pons neurons, with slightly higher levels in LC neurons of donor P5 and P7. (**7**) *Rubidium* levels at 1–2 ppm were present in many neurons of the LC and anterior pons, but absent in some LC neurons, for example in donor P6.

Low levels of arsenic, cobalt, gold, chromium, manganese, nickel, platinum, and titanium were seen in a few LC neurons in some donors. In three donors an occasional LC neuron with titanium levels above 2 ppm was observed. These results, as well as the intraneuronal levels of calcium, chloride, phosphorous, sulfur and zinc, are provided in the **S1 Table**.

## Discussion

Key findings of this study are that human LC neurons contain variable concentrations of a variety of toxic metals and essential trace elements. These results suggest that high levels of toxic metals, or low or high levels of essential trace elements, in individual LC neurons could lead to a decline of noradrenaline output to focal regions of the CNS innervated by these neurons. This loss of noradrenaline could trigger a variety of neurodegenerative or demyelinating disorders, and could determine the CNS topography of these widespread multifocal disorders.

Either high or low levels of most of the LC-variable elements we found have been implicated in neurodegenerative disorders [35–40]. The tissue we examined was from people with multiple sclerosis, and variations in levels of some of these elements have also been implicated in this condition. (**1**) *Mercury* can cause the autoimmune, inflammatory, cytotoxic, genetic and epigenetic changes [40] that have been implicated in multiple sclerosis [41]. Mercury first appears in the LC starting from about 20 years [20], an age at which multiple sclerosis often first presents clinically. Mercury can disturb cellular mechanisms without causing cell loss [27], which may explain why LC damage, but not cell loss, is described in multiple sclerosis [17]. (**2**) In LC neurons that contained mercury, high *selenium* levels were present, in a 1:1 molar ratio, implying selenium was bound to mercury, a common method of protection from mercury toxicity [42]. Selenium has been proposed to play a role in several chronic diseases of the nervous system [43], and selenium treatment could modulate mercury-induced neurotoxicity [44]. It has been suggested that mercury toxicity may be due to a selenium deficiency caused by mercury binding, which leaves insufficient selenium available to function as a component of vital enzymes [45]. Low selenium levels could also result in increased oxidative stress due to inhibition of the antioxidative properties of some of the more than 30 selenium-dependent proteins, such as glutathione peroxidase [43]. Although selenium levels in our LC neurons appeared adequate to bind mercury, it remains possible that during the period of initial mercury exposure selenium levels were low, due either to inadequate selenium intake, or to the presence of genetic polymorphisms that affect selenium status [46]; the presence of these polymorphisms in people with multiple sclerosis may warrant future investigation. Decreased blood levels of selenium have been described in a small number of multiple sclerosis patients [47], but studies of larger numbers would be needed to confirm this. (**3**) *Iron* has a role in generating oxygen free radicals [48] and has long been suspected to play a part in the pathogenesis of multiple sclerosis [49,50]. Polymorphisms in genes for iron regulation have been associated with multiple sclerosis [51], and progressive multiple sclerosis has been linked to age-related increases in CNS iron [52,53]. (**4**) Alterations in *copper* levels have been implicated in multiple sclerosis [54]. A chelator-induced copper deficiency can trigger demyelination in a mouse model [55,56]. (**5**) An association of multiple sclerosis with exposure to *lead* in soil has been reported [57]. (**6**) *Organobromine* compounds, commonly found in fire retardants, as well as in pharmaceuticals and historically in pesticides, can cross the blood-brain barrier, accumulate in the brain, and adversely affect neuronal functions [37]. It would be of interest to trace the origins of the bromine present in LC neurons by conducting speciation tests using another synchrotron technique, X-ray absorption spectroscopy. (**7**) *Rubidium* has no known biological functions, but is normally present in most cells [58]. Low tissue levels of rubidium are found in tissue homogenates from Alzheimer’s disease brains, possibly due to impaired intracellular energy production that causes decreased ion transport of both potassium and rubidium (which can be exchanged with potassium); it has been suggested that rubidium could be used as a proxy for potassium in imaging the brain to detect impaired energy production [58]. We found some LC neurons contained abundant potassium but no detectable rubidium, so in the LC the link between rubidium and potassium appears to be less clear.

The routes of human exposure for most of the individual toxic metals that differed among LC neurons, and their varied mechanisms of toxicity within the nervous system, are well described [59,60]. An advantage of synchrotron XFM is that it can simultaneously measure the levels of many different toxic and essential elements within the same cell [25]. The value of multi-element analyses is becoming clear with the realisation that damaging synergistic interactions between toxic metals are common [59–63]. The two mechanisms that appear to be particularly augmented by mixtures of toxic metals are the generation of reactive oxygen species and interference with the functions of essential metals [60], both mechanisms suspected to play a part in many neurological disorders, including multiple sclerosis [54,64].

The reason the LC selectively takes up a variety of toxic metals, especially mercury, is not yet understood, though several possibilities exist [20]. (**1**) Neuromelanin starts appearing in LC neurons at an early age, possibly as a protective factor against metal toxicity [65]. Binding of toxic metals to neuromelanin may retain these metals in the cell indefinitely, whereas most other cell types have mechanisms to remove mercury [66]. This has led to the suggestion that analysis of the metal content of LC neurons could be an indicator of past exposure to these metals [67]. The substantia nigra in the midbrain also contains neuromelanin, and though toxic metals bind to nigral neuromelanin [68], mercury uptake by the LC appears to be more marked than uptake by the substantia nigra [67]. This lends weight to the ‘locus ceruleus-first’ hypothesis to explain the loss of substantia nigra neurons in Parkinson’s disease [12], which posits that an early LC neuronal loss in Parkinson’s disease [69], possibly due to the selective uptake of toxic metals, leads to a lack of noradrenaline neuroprotection of substantia nigra neurons [70], which then succumb to the neurotoxic effects of their normally-high iron levels [39]. (**2**) The LC supplies noradrenaline to microvessels in the brain and spinal cord, and so has a potential exposure to circulating toxins. Retrograde axonal transport of toxic metals to the LC neuronal cell bodies could result in accumulation of these toxicants [21]. (**3**) Metal transporters are responsible for the selective uptake of metals such as mercury, cadmium, iron, and manganese in several different cell types [71–73]. Future studies of the cellular distribution of these metal transporters in LC neurons could shed light on their role in the selective uptake of metals. (**4**) Other suggestions as to why the LC accumulates toxicant selectively are that it has a rich blood supply, and that it could be exposed to toxins in the cerebrospinal fluid of the adjacent fourth ventricle [20].

Future studies to test the hypothesis of toxicant-induced neurodegeneration could overcome the limitations of the present study. (**1**) Our finding that similar proportions of people with and without multiple sclerosis have mercury in their LC neurons implies that it is likely people from other backgrounds will also have variations in a variety of LC elements. Elemental analysis of the LC in a wider range of conditions, including people with no neurodegenerative disorders, would be able to confirm this. It would, however, be difficult to analyse people who have died after a long history of Alzheimer’s or Parkinson’s disease, since at advanced stages of these diseases LC neuronal loss is severe. Unfortunately, no non-invasive methods to detect intraneuronal elemental concentrations early in the course of neurodegenerative conditions are currently available. (**2**) The LC is a long thin rostro-caudal collection of neurons but we were only able to sample it at one horizontal level since this was a retrospective study of routinely-sampled tissue. Elemental analyses at several different horizontal levels of the LC could be revealing, since rostro-caudal topographical differences in LC neuron loss, of unknown cause, are described in Alzheimer’s disease, Parkinson’s disease, and normal aging. (**3**) We examined LC and anterior pons neurons, but other neuronal collections in the brain and spinal cord could have similar elemental variations among individual neurons. For example, XFM could be used to measure elements in spinal motor neurons to see if variations could underlie the susceptibility of certain subpopulations of motor neurons to amyotrophic lateral sclerosis [74], especially since it is known that in this disorder mercury is present in only some LC and motor neurons [75,76]. (**4**) Only a proportion of people with multiple sclerosis had mercury in their LC neurons, so it is possible that a variety of non-mercury toxic metals could damage these neurons, with individual genetic susceptibilities to each toxicant responsible for disease onset. To test this, whole exome or whole genome analyses could be undertaken (on blood samples taken before death, or from autopsied CNS tissue) and the genotypes compared with the toxic metals found in the LC on autopsy; numerous polymorphisms have now been described that affect the metabolism of metals such as mercury [77] and iron [51].

In conclusion, variations in concentrations of toxic metals and essential trace elements are found among human LC neurons, and these elemental concentrations vary between people. These variations may underlie the damage detected in LC neurons in a variety of neurodegenerative and demyelinating disorders. Further studies of elemental concentrations in individual neurons, in a wide variety of clinicopathological conditions, in different sites in the CNS, and combined with genetic susceptibility studies, are likely to shed further light on the role of toxic and essential elements in these disorders.

## Supporting information

S1 Fig Example of generation of histograms from elemental mapping. Six neurons sampled from the locus ceruleus of donor P2 are circled in a high-resolution potassium map (**A**) with corresponding histograms showing the elemental concentrations (**B**) indicate that neurons 1, 2 and 3 have high mercury and selenium levels, compared to the other three neurons, whereas neuron 5 has a high iron level. Six neurons circled from the paired anterior pons (**C**) with corresponding histograms (**D**) indicate neurons 1 and 2 have higher iron levels than the other four neurons; none contains mercury. BV: lumen of a blood vessel, surrounded by endothelial cells. Measurements are normalised to 1000 ppm of potassium. Colour bar = relative concentrations of elements in parts per million (ppm) (the absolute concentrations can be viewed in **S1 Table**). *The histograms from donor P2 are repeated in Fig 5 to facilitate comparisons with other donors.*
(TIF)

S1 Table Elemental concentrations in individual neurons. Supplementary Data Sheets 1 to 7 show concentrations of all elements in individual neurons from patients P1 to P7, as well as statistics for concentrations averaged from multiple neurons (‘bulk’), uncertainty estimates (‘error bars’), and minimum detectable levels (‘MDL’). AP: anterior pons, LC: locus ceruleus.
(XLSX)
