# Supplementary figures and images for: Concentrations of toxic metals and essential trace elements vary among individual neurons in the human locus ceruleus
Source: PLoS One. 2020 May 19;15(5):e0233300. doi: 10.1371/journal.pone.0233300 (PMC7237016; doi:10.1371/journal.pone.0233300)

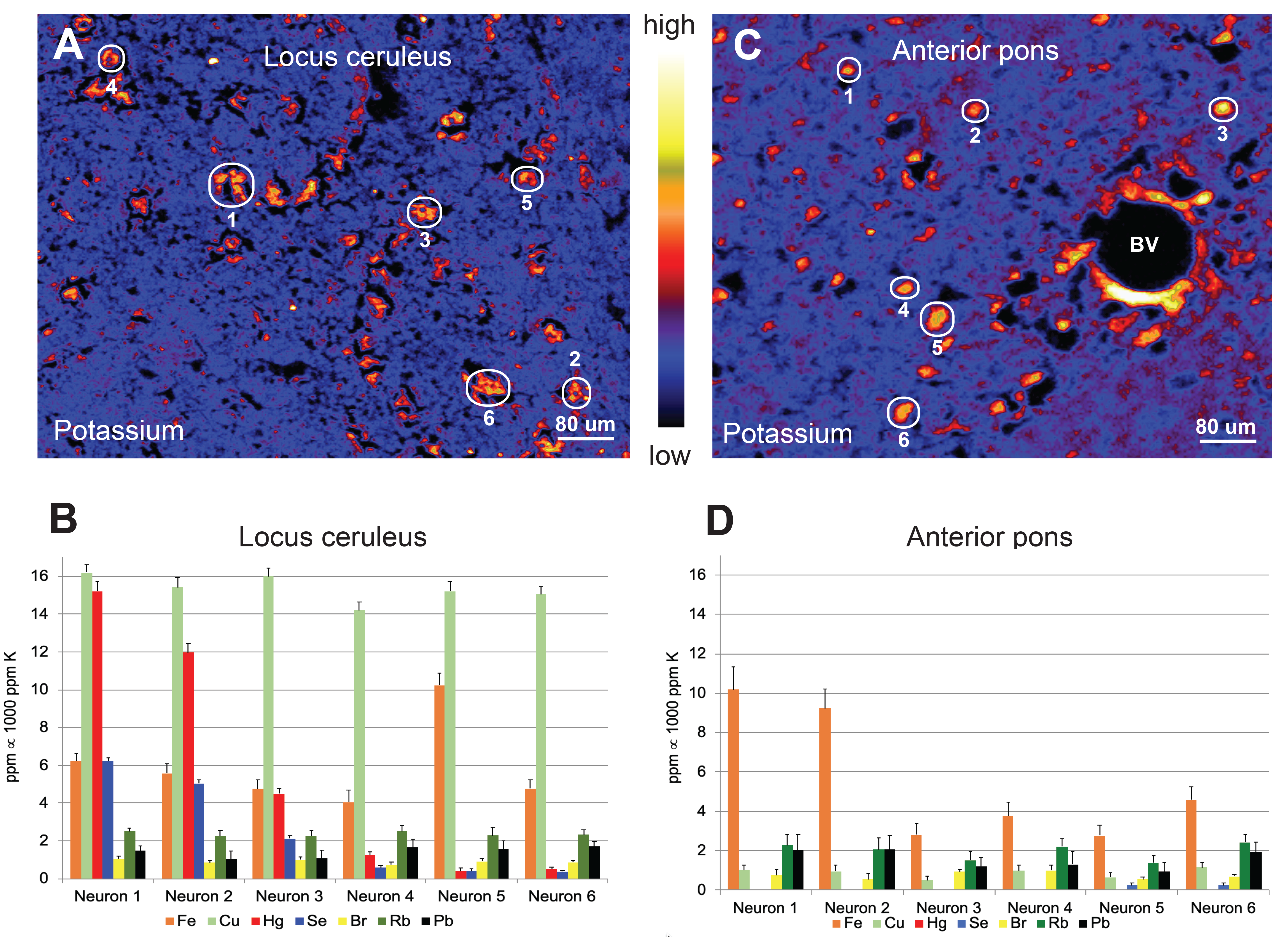

Supplement: S1 Fig — Six neurons sampled from the locus ceruleus of donor P2 are circled in a high-resolution potassium map (A) with corresponding histograms showing the elemental concentrations (B) indicate that neurons 1, 2 and 3 have high mercury and selenium levels, compared to the other three neurons, whereas neuron 5 has a high iron level. Six neurons circled from the paired anterior pons (C) with corresponding histograms (D) indicate neurons 1 and 2 have higher iron levels than the other four neurons; none contains mercury. BV: lumen of a blood vessel, surrounded by endothelial cells. Measurements are normalised to 1000 ppm of potassium. Colour bar = relative concentrations of elements in parts per million (ppm) (the absolute concentrations can be viewed in S1 Table). The histograms from donor P2 are repeated in Fig 5 to facilitate comparisons with other donors. (TIF) [file pone.0233300.s001.tif]
